# Subwavelength Quasi-Periodic Array for Infrared Antireflection

**DOI:** 10.3390/nano12193520

**Published:** 2022-10-08

**Authors:** Haoran Wang, Fan Zhang, Ji’an Duan

**Affiliations:** 1 State Key Laboratory of High Performance Complex Manufacturing, Central South University, Changsha 410083, China; 2 School of Automation, Central South University, Changsha 410083, China

**Keywords:** infrared antireflection, femtosecond laser, double-pulse, infrared imaging

## Abstract

Infrared antireflection of a zinc sulfide (ZnS) surface is important to improve performance of infrared detector systems. In this paper, double-pulse femtosecond laser micro-machining is proposed to fabricate a subwavelength quasi-periodic array (SQA) on ZnS substrate for infrared antireflection. The SQA consisting of approximately 30 million holes within a 2 × 2 cm^2^ area is uniformly formed in a short time. The double-pulse beam can effectively suppress the surface plasma shielding effect, resulting in obtaining a larger array depth. Further, the SQA depth is tunable by changing pulse energy and pulse delay, and can be used to readily regulate the infrared transmittance spectra as well as hydrophobicity. Additionally, the optical field intensity distributions of the SQA simulated by the rigorous coupled-wave analysis method indicate the modulation effect by the array depth. Finally, the infrared imaging quality captured through an infrared window embedded SQA is evaluated by a self-built infrared detection system.

## 1. Introduction

Zinc sulfide (ZnS) is a widely utilized infrared material with stable chemical and physical properties for infrared window application especially in harsh and thermal shock environments [1]. However, the high refractive index of ZnS (*n* = 2.2 at 10.6 µm) generally induces large surface Fresnel reflection losses (about 25%), influencing the imaging quality of the infrared detection system [2,3]. Accordingly, antireflection on ZnS can effectively suppress surface Fresnel reflection, and is vital to increase surface transmittance for the application of infrared imaging, unmanned surveillance, and infrared guidance [4,5]. The conventional method to realize antireflection is to coat with a multilayer dielectric film, which suffers the shortage of easy exfoliation due to the mismatch of thermal expansion and crystal lattice between the ZnS substrate and surface coating [6,7]. An alternative approach to eliminate surface reflection is directly patterning antireflective surface structures on ZnS with advantages of lattice adaptation, polarization insensitivity, and wide acceptance angles [8,9]. In order to flexibly fabricate antireflective structures for infrared window application, several methods such as reactive ion etching [10], chemical etching [11], interference lithography [12], and nanoimprinting [13] have been reported, of all which suffer from some limitations, for example, being environmentally-unfriendly, high-cost, multi-step, and time-consuming.

Recently, femtosecond laser micromachining has become a promising tool to produce highly precise antireflective structures in one-step processing due to its advantages of it having less heat-zone, and being mask-less, non-chemical, and environmentally-friendly [14,15,16,17]. Bushunov et al. fabricated antireflection microstructures by single-pulse femtosecond laser to increase surface transmittance [18]. Li et al. proposed femtosecond laser Bessel beam to ablate concave antireflective subwavelength structures on ZnS, achieving an increase in transmittance of 5.7% at 7.2 μm wavelength [19]. Chen et al. presented a femtosecond laser direct writing method to produce subwavelength antireflective surfaces with high and broadband transmittance [20]. Duan et al. proposed a femtosecond laser parallel multi-beam to rapidly form subwavelength antireflective microstructures on ZnS, achieving broadband response and a large acceptance angle as well as robust mechanical strength and hydrophobicity [21]. Wang et al. [22] designed and fabricated an antireflective subwavelength structure by femtosecond laser Bessel beam, resulting in transmittance enhancement in the far-infrared band. However, the depth and quality of antireflective structures are often limited by the surface plasma shielding effect [23,24], which therefore influences the antireflective performance of the infrared window.

In this paper, a double-pulse femtosecond laser is used to fabricate a subwavelength quasi-periodic array (SQA) on the ZnS substrate for infrared antireflection. The double-pulse beam can effectively suppress the surface plasma shielding effect, resulting in obtaining a larger array depth. Moreover, the array depth is regulated by pulse energy and pulse delay, and is utilized to adjust transmittance spectra and wettability of SQA. Consequently, the infrared imaging quality captured through an infrared window embedded SQA is analyzed by a self-built infrared detection system.

## 2. Experiment and Simulation

**Figure 1:**
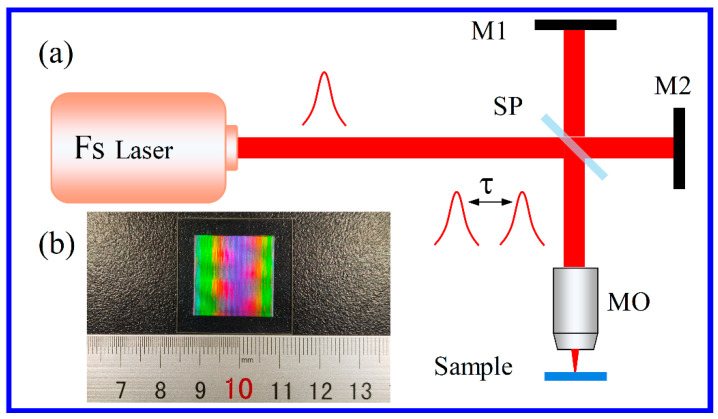
(**a**) Experimental optical system for femtosecond laser double-pulse beam fabrication. Fs: femtosecond, SP: 5/5 spectroscope, M1 and M2: mirror, MO: objective lens, τ: pulse delay. (**b**) Photograph of the laser treated sample.

**Figure 2:**
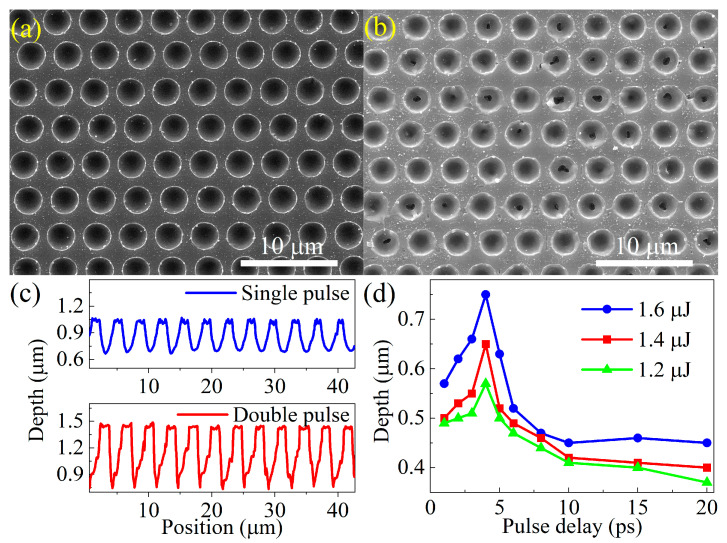
SEM images of the SQA fabricated by single-pulse (**a**) and double-pulse (**b**) femtosecond laser, and corresponding cross section profiles (**c**). (**d**) Depth of the SQA as a function of pulse delay with total pulse energy of 1.6 μJ, 1.4 μJ, and 1.2 μJ.

A commercial ZnS sample of size 30 mm × 30 mm × 2 mm fabricated by chemical vapor deposition method was fixed on a three-dimensional motion platform (Newport) with precision of 20 nm [25]. A regenerative amplified Yb: KGW femtosecond laser (Pharos, Light Conversion) with a central wavelength of 1030 nm, pulse duration of 216 fs, and repetition rate of 10 kHz was used to generate a Gaussian beam. A Michelson interferometer setup was employed to temporally shape the pulses into a double-pulse beam with the energy ratio of 1:1, as shown in Figure 1a. The pulse delay Δτ = ΔL/c is controlled by adjusting the optical path length ΔL through a linear translation stage, where c is light speed. The two-pulse sequence is conveyed through the Newport workstation, equipped with a motorized nanometric resolution stage allowing the sample precise positioning. The double-pulse energy could be adjusted through a neutral density attenuator and was set to 1.6 μJ. Then, a 40× objective lens (numerical aperture = 0.6) was employed to focus the incident laser beam on the ZnS sample surface at normal incidence. The ZnS sample was translated in the X direction vertical to the laser beam at a speed of 35 mm/s, resulting in ablation holes with separation of 3.5 μm at laser repetition ratio of 10 kHz. In order to generate a hole array, a square was produced along the Y direction with a 3.5 μm transverse distance between scanning lines. However, the hole distance between up line and down line is equal to or larger than the value of 3.5 μm, as shown in Figure 2a,b. Therefore, the SQA is not completely periodic, and is named quasi-periodic.

Finally, the surface morphologies and profiles of the prepared samples can be successively measured by a scanning electron microscope (SEM, Tescan, Brno, Czechia) and a confocal laser scanning microscope (CLSM, Zeiss, Oberkochen, Germany). The infrared transmittance spectra and wettability of the ZnS were obtained by an infrared microscope (FTIR, Thermo Fisher, Waltham, MA, USA) and an optical contact angle meter (Harke, Beijing, China), respectively. The infrared target images were achieved by a self-built infrared detection system with components of thermal detector (FLIR, Wilsonville, OR, USA) and infrared window.

In simulation, the SQA was designed to accommodate the fabricated samples, to explore the antireflective mechanism in the wavelength of 9 μm by using the rigorous coupled-wave analysis (RCWA) method. The direction of the incident beam propagation (Z direction) and the other two vertical directions of beam propagation (X and Y directions) were set by the transmission-line treatment and the periodic boundary condition, respectively. The grid mesh size with 20 nm provides a high resolution for obtaining accurate results. The simulated time was chosen as 1000 fs to obtain a stable electric field intensity distribution for the designed SQA.

## 3. Results and Discussion

Figure 1b depicts the prepared SQA of area 2 × 2 cm^2^ consisting of approximately 30 million holes on the ZnS substrate. The multiple rainbow patterns under white light illumination are observed on the sample surface, indicating that the irradiated holes are effectively separated without overlapping. The total manufacturing process only takes about 50 min, indicating that the laser scanning approach has the ability to form a large-area SQA on ZnS.

Figure 2a,b shows the surface morphology of fabricated SQA on ZnS by single-pulse and double-pulse femtosecond lasers, respectively. It can be observed that the laser-induced holes with diameter of 2.6 μm are quasi-regularly arranged on the surface of the substrate, which is attributed to the insufficient accuracy of the vibrated motion platform during the fast process of laser irradiation [26]. Consequently, the quasi-periodic array is formed on ZnS with a 3.5 μm period and 43.3% fill factor, which satisfies the subwavelength demand in the infrared band [27]. Moreover, the average surface roughness of the SQA is 0.134 μm within a 42 × 42 μm^2^ scanning region, expressing a good flatness as demonstrated from the CLSM measurement. It can effectively reduce surface scattering and enhance transmittance of the ZnS surface. Figure 2c exhibits the CLSM-measured cross-sectional profiles of the SQAs, fabricated by single-pulse and double-pulse femtosecond laser. The SQA depth from double-pulse irradiation is 0.71 μm, which is almost twice as much as that from single-pulse irradiation with a value of 0.34 μm. Therefore, the increase in pulse number effectively increases the irradiated hole depth, which is attributed to suppressing the surface plasma shielding effect during crater ablation by femtosecond laser [28]. Further, the increase of SQA depth is beneficial to improve the antireflective performance in the infrared band [29].

In order to explore the evolution of SQA depth by double-pulse irradiation, the pulse delay dependent array depth within the range of 1–20 ps is shown in Figure 2d. Apparently, the SQA depth increases for the measured pulse delay as pulse energy enhances from 1.2 μJ to 1.6 μJ, originating from the greater generation of color centers and stronger micro-explosion [30]. The SQA depth sharply increases from 0.56 μm to 0.75 μm with pulse delay increasing from 1 ps to 4 ps for the pulse energy of 1.6 μJ. Subsequently, a declining trend in SQA depth evolution is observed with a longer pulse delay in 4–20 ps. The double-pulse laser ablation of ZnS substrate experiences a nonlinear process with avalanche and multiphoton ionization, and leads to fast transient modification in pulse duration [31]. The first pulse is the preconditioning pulse, and the second pulse availably triggers ablation. In addition, considering that the absorption coefficient of ZnS is inversely proportional to the refractive index [32], the refractive index of ZnS decreases with increasing temperature, resulting in enhanced energy absorption of the second pulse on ZnS after the first pulse irradiation. However, during the longer pulse delay, the weaker melting induced by the low first pulse laser energy results in the following pulse producing hardly considerable ablation.

**Figure 3:**
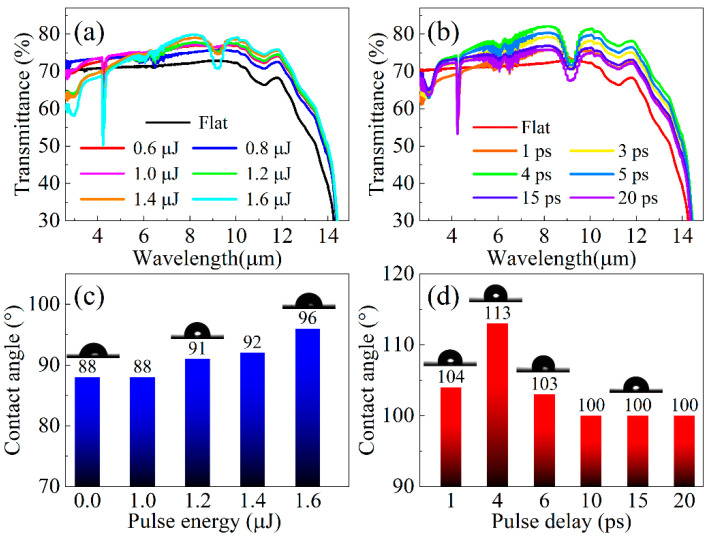
Measured infrared spectra of SQA fabricated by the double-pulse beam for different pulse energies (**a**) and pulse delay (**b**); and measured contact angle of the water droplet on ZnS fabricated by double pulse femtosecond laser at different pulse energy (**c**) and pulse delay (**d**).

Figure 3a illustrates the infrared spectra of the SQA at different pulse energies for pulse delay of 0 ps in the infrared band. It can be seen that the transmittances of SQA are larger than those of flat ZnS for the measured band. Moreover, the maximum transmittance values for the SQA are located around 8.1 μm wavelength, due to the subwavelength feature of the 3.5 μm period from double-pulse laser microfabrication. In particular, the largest SQA transmittance for 1.6 μJ pulse energy reaches 80.1% compared with that of 72.5% in the flat ZnS, proving that surface Fresnel reflection is suppressed by introducing SQA. Furthermore, the transmittance values of SQA increase with pulse energy enhancing from 0.6 μJ to 1.6 μJ in the 6–14 μm wavelength. The depth enhances with increasing pulse energy, more effectively inducing a smoothly graded dielectric layer on ZnS against surface Fresnel reflection. Figure 3b demonstrates that the infrared transmittances of SQA first increase then decrease with pulse delay enlarging from 1 ps to 20 ps at pulse energy of 2 μJ, which is in accord with the evolution of array depth in Figure 2d. Therefore, the antireflective performance of SQA is able to be tuned by changing the pulse delay in double-pulse femtosecond laser micro-fabrication.

The wettability of the SQA is an important feature of the infrared window in rainy and foggy weather, since surface droplets can influence the antireflective performance and infrared imaging. Figure 3c,d shows the water contact angles and corresponding optical images on surfaces at different pulse energy and pulse delay. The contact angle of the flat ZnS with value of 88° is smaller than that of the SQA, demonstrating that the hydrophobicity of surface ZnS is significantly improved by the fabricated SQA. In addition, when pulse energy increases from 1.0 μJ to 1.6 μJ, the contact angle of the SQA increases from 88° to 96° at pulse delay of 0 ps for double-pulse femtosecond laser irradiation. The contact angle first increases then decreases with pulse delay enhancing from 1 ps to 20 ps at pulse energy of 2 μJ, as exhibited in Figure 3d. These tendencies for contact angle are achieved in accordancewith the evolution of array depth by adjusting pulse energy and pulse delay. The mechanism lies in the rougher surface induced by deeper depth enhancing the surface hydrophobicity following the Wenzel model [33]. Consequently, the SQA formed on ZnS can be applied in extreme weather usually prohibitive for the infrared window, due to its hydrophobic, self-cleaning, and anti-fog properties.

**Figure 4:**
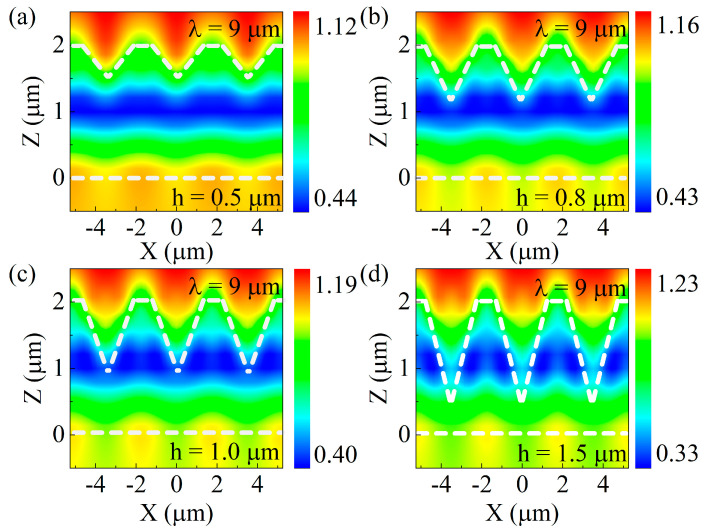
Simulated electric field intensity distributions for SQA with period of 3.5 μm and heights of (**a**) 0.5 μm, (**b**) 0.8 μm, (**c**) 1.0 μm, and (**d**) 1.5 μm in 9 μm wavelength.

The antireflective mechanism can be elucidated from the interaction between detected infrared light and the SQA, and therefore the electric field (light field) intensity distributions are calculated on the SQA with different heights in 9 μm wavelength, as depicted in Figure 4. The infrared light intensity is mostly immersed in the SQA instead of the outside environment. The laser-ablated structures on the surface of ZnS can capture the detected incident infrared light. Specifically, the photons violently collide with and reflect off the sidewalls, resulting in local light field enhancement (LLFE) [34,35]. Inversely, incident infrared photons are also captured, which can effectively reduce surface Fresnel reflection on ZnS. Moreover, as the array heights increase from 0.5 μm to 1.5 μm, the LLFE in the SQA becomes more significant. The larger height is able to restrict more photons in the SQA, promoting the antireflective performance on the ZnS infrared window.

**Figure 5:**
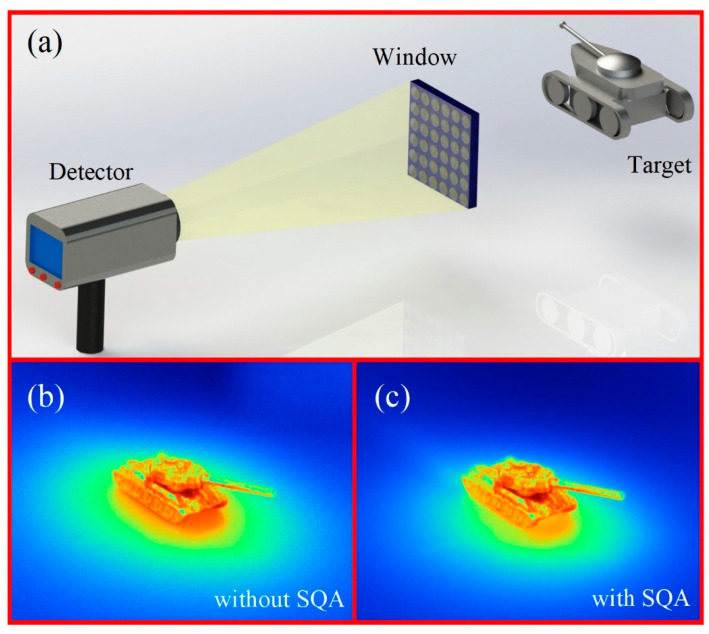
(**a**) Schematic diagram of the self-built infrared detection system consisting of target, infrared window, and infrared detector: the measured target images at temperature of 303 K through infrared window without (**b**) and with (**c**) SQA.

In order to explore infrared imaging quality through fabricated SQA, an infrared detection system including detector, window, and target is carefully built, as shown in Figure 5a. The infrared window in front of the infrared detector is a ZnS substrate with SQA fabricated by double-pulse femtosecond laser, with pulse energy of 1.6 μJ and pulse delay of 4 ps. Figure 5b,c show the captured infrared image from infrared window without and with SQA. It can be observed that the SQA enhances the image details and suppresses the background noise at measured temperature of 303 K, which corresponds to incident wavelength of 9.56 μm. The calculated mean pixel contrast (MPC) index of the infrared image captured through SQA is enhanced from 26 (without laser treatment) to 38 (with). The underlying reason is attributed to the infrared transmittance enhancement of infrared window with the fabricated SQA, promoting greater capture of infrared signal by the infrared detector. The SQA can effectively increase the transmittance of infrared window and suppress surface Fresnel reflection. The effective signals captured by the infrared detector are enhanced through the infrared window. However, most of the image noise comes from self-heating excitation in the infrared detector. Therefore, the signal-noise ratio of the infrared detection system increases by introducing SQA. In addition, we can list the mean pixel contrast index enhancement between our method and antireflective subwavelength structures (ASS) in [22], and the results are shown in Table 1. The SQA has apparent large contrast enhancement with a value of 46.1% compared to that of 32.3% for ASS, which demonstrates the superiority of our method. Moreover, for processing samples of size 2 × 2 cm^2^, the double-pulse method takes 50 min, which is less than that of the reference [22] by about 2.8 h.

## 4. Conclusions

In summary, a temporarily-shaped femtosecond laser double-pulse beam is employed to fabricate SQA on ZnS to increase its transmittance. The SQA with period of 3.5 μm is uniformly formed on a 2 × 2 cm^2^ area within 50 min. In addition, the array depth can be readily regulated through pulse energy and pulse delay, which is attributed to suppressing the surface plasma shielding effect during crater ablation. The obtained SQA achieves broadband antireflection with tunable transmittances in the infrared band as well as controlled hydrophobicity, which is in accord with the evolution of array depth. The infrared detection measurements at 9.56 μm incident wavelength indicate that the SQA can effectively improve the clarity of the captured infrared image. The proposed fabrication method for an antireflective surface can be applied in infrared target tracking.

## Figures and Tables

**Table 1:** Mean pixel contrast index of SQA and ASS.

Sample	Period	Processing Time	MPC Enhancement
SQA	3.5 µm	50 min	46.1%
ASS	3.8 µm	2.8 h	32.3%

## Data Availability

The study did not report any data.
